# Evaluation of a training program of hypertension for accredited social health activists (ASHA) in rural India

**DOI:** 10.1186/s12913-018-3140-8

**Published:** 2018-05-02

**Authors:** Marwa Abdel-All, Amanda Gay Thrift, Michaela Riddell, Kavumpurathu Raman Thankappan Thankappan, Gomathyamma Krishnakurup Mini, Clara K. Chow, Pallab Kumar Maulik, Ajay Mahal, Rama Guggilla, Kartik Kalyanram, Kamakshi Kartik, Oduru Suresh, Roger George Evans, Brian Oldenburg, Nihal Thomas, Rohina Joshi

**Affiliations:** 1 The George Institute for Global Health, et Sydney, PO Box M 201, Missenden Road, Camperdown, New South Wales 2050 Australia; 2 Sydney Medical School, University of Sydney, Sydney, New South Wales Australia; 3 Department of Medicine, School of Clinical Sciences at Monash Health, Monash University, Melbourne, Victoria Australia; 4 Achutha Menon Centre for Health Science Studies, Sree Chitra Tirunal Institute for Medical Sciences and Technology, Trivandrum, Kerala India; 5 Amrita Institute of Medical Sciences, Kochi, Kerala India; 6 Western Sydney Local Health District, Westmead Hospital, Westmead, Australia; 7 The George Institute for Global Health, New Delhi, India; 8 The George Institute for Global Health, Oxford University, Oxford, UK; 9 Melbourne School of Population and Global Health, University of Melbourne, Melbourne, Victoria Australia; 10 Nossal Institute for Global Health, Melbourne School of Population and Global Health, University of Melbourne, Melbourne, Victoria Australia; 11 Rishi Valley Rural Health Centre, Rishi Valley, Andhra Pradesh India; 12 Cardiovascular Disease Program, Biomedicine Discovery Institute and Department of Physiology, Monash University, Melbourne, Victoria Australia; 13 Department of Endocrinology, Diabetes and Metabolism, Christian Medical College, Vellore, Tamil Nadu India; 14 Faculty of Medicine, University of New South Wales, Sydney, New South Wales Australia

**Keywords:** ASHA, Training evaluation, India, Hypertension, Kirkpatrick evaluation model

## Abstract

**Background:**

Hypertension is a major risk factor for cardiovascular disease, a leading cause of premature death and disability in India. Since access to health services is poor in rural India and Accredited Social Health Activists (ASHAs) are available throughout India for maternal and child health, a potential solution for improving hypertension control is by utilising this available workforce. We aimed to develop and implement a training package for ASHAs to identify and control hypertension in the community, and evaluate the effectiveness of the training program using the Kirkpatrick Evaluation Model.

**Methods:**

The training program was part of a cluster randomised feasibility trial of a 3-month intervention to improve hypertension outcomes in South India. Training materials incorporated details on managing hypertension, goal setting, facilitating group meetings, and how to measure blood pressure and weight. The 15 ASHAs attended a five-day training workshop that was delivered using interactive instructional strategies. ASHAs then led community-based education support groups for 3 months. Training was evaluated using Kirkpatrick’s evaluation model for measuring reactions, learning, behaviour and results using tests on knowledge at baseline, post-training and post-intervention, observation of performance during meetings and post-intervention interviews.

**Results:**

The ASHAs’ knowledge of hypertension improved from a mean score of 64% at baseline to 76% post-training and 84% after the 3-month intervention. Research officers, who observed the community meetings, reported that ASHAs delivered the self-management content effectively without additional assistance. The ASHAs reported that the training materials were easy to understand and useful in educating community members.

**Conclusion:**

ASHAs can be trained to lead community-based group educational discussions and support individuals for the management of high blood pressure.

**Trial Registration:**

The feasibility trial is registered with the Clinical Trials Registry - India (CTRI) CTRI/2016/02/006678 (25/02/2016).

**Electronic supplementary material:**

The online version of this article (10.1186/s12913-018-3140-8) contains supplementary material, which is available to authorized users.

## Background

Cardiovascular disease (CVD) is the leading cause of premature death and disability in India, predicted to reach up to 2.6 million deaths by 2020 [1]. Hypertension, a major risk factor for CVD, is responsible for 57% of all deaths from stroke and 24% of all deaths from coronary heart disease in India [2]. About 33% of urban and 25% of rural Indians have hypertension but less than a third are aware of their status and have their blood pressure under control [3]. Populations residing in rural regions of India face several challenges in accessing care for CVD. These include poor knowledge of risk factors, lack of physicians and nurses, and unavailability of affordable medicines [4]. Prevention and control of hypertension can be enhanced at the individual level by improving knowledge of hypertension and risk factors, adoption of a healthy lifestyle, and adherence to medications [5, 6]. At the health system level, hypertension can be controlled by having adequate number of healthcare providers, provision of evidence-based management guidelines or decision support tools, improving the availability of medicines and access to health centres. When physicians and nurses are in short supply, tasks such as screening, education, referral and follow-up of individuals in the community can be shifted to non-physicians [7] such as community health workers (CHWs) [8, 9]. CHWs enhance coverage of essential healthcare services to the community [10], are cost-effective and are usually well accepted by the communities [11]. The Indian government launched the National Rural Health Mission (NRHM) in 2005 and created a new cadre of non-physicians called Accredited Social Health Workers (ASHAs) [12]. ASHAs are India’s CHWs who act as an interface between the community and the public health system to improve access to health services, help raise community awareness about health and its social determinants and support the primary healthcare system in facilitating care specifically for maternal and child health [13]. They are female residents of the villages [14] who are paid a fee-for service for certain primary health care activities [15]. Previous evaluation studies of ASHAs have mostly focused on maternal and child care [16, 17]. Since the inauguration of the National Program for the Prevention and Control of Cardiovascular Diseases, Diabetes, Cancer and Stroke (NPCDCS) in 2010, ASHAs from 100 pilot districts across 21 States have been trained by the public health system to prevent and manage chronic diseases like hypertension, diabetes and their risk factors. The current study of control of hypertension in rural India was conducted at three sites, one of which was a pilot NPCDCS site. The objectives were to (i) develop training materials for an intervention to improve identification and control of hypertension, (ii) document the processes of training ASHAs, and (iii) evaluate the effectiveness of the training program in terms of the knowledge, skills and perception of ASHAs using the Kirkpatrick Evaluation Model [18].

## Methods

### Area settings and recruitment

This study was part of a trial aimed at improving self-management and control of hypertension in rural India (CHIRI) [19]. The main study was conducted to investigate whether populations at various stages of economic transition, with different levels of knowledge and awareness of hypertension, and with differing barriers to diagnosis and treatment of hypertension, could have their hypertension managed using a community-based education program. The late transition populations are characterized by rapid urbanization and a sedentary lifestyle. They will have higher prevalence of cardiovascular disease and its risk factors like hypertension and diabetes [20]. They also have relatively better access to health services and greater health awareness. On the contrary, early transition populations are economically disadvantaged with poor access to health services and have a lower socio-economic status. The study was conducted in three regions in South India, Trivandrum-Kerala (late transition), West Godavari-Andhra Pradesh (medium transition) and Rishi Valley-Andhra Pradesh (early transition) [19]. The outcome of the feasibility trial will be reported in due course.

During the first phase of the study, a cross-sectional survey was conducted amongst local community members to determine the knowledge, awareness, treatment and control of hypertension in these regions. This was supplemented by an audit of the health system and a qualitative study to understand the major barriers in the management of hypertension [19]. Based on the information gathered during this first phase, a community-based group intervention was developed and its feasibility was tested at the three sites. The intervention study comprised group-based education and support for self-management of blood pressure in individuals with hypertension. Individuals with hypertension could also invite their family members to attend, if they wished. At the community level, the intervention was led by ASHAs.

### Curriculum development and training

Content of the training materials (please see Additional file 1: Table S1) was created by the research team based on available literature, experience from relevant projects such as the Australasian Peers for Progress Diabetes Project, Kerala Diabetes Prevention Program (K-DPP) [21], the Andhra Pradesh Rural Health Initiative [22], and the available ASHA Training Modules [23]. Training materials were translated into the local languages (Telugu and Malayalam versions will be provided online) and a team of local research staff and clinicians reviewed the training materials to ensure it was culturally adapted to the local context. Content validity of the training materials was assessed by piloting it with four ASHAs from a non-study village that led to further modification and refinement of the resources.

Training was designed to provide ASHAs with knowledge regarding hypertension and its risk factors, strategies to manage hypertension via knowledge about healthy lifestyle and adherence to medications, and skills in facilitation of group meetings. ASHAs were also taught to deliver community group-based education, and provide support for individuals with hypertension.

Based on previous experience and available literature, five main themes were addressed during the training sessions. These included:

1. Hypertension, its risk factors, complications, management and clinical targets.
2. Healthy lifestyles and how to support the community to adopt a healthier lifestyle.
3. Goal-setting strategies and how to assist group members to set and achieve simple specific, measurable, achievable, realistic/relevant and time bound (SMART) goals to improve health outcomes.
4. Skills needed for taking clinical and anthropometric measures such as weight and blood pressure.
5. Group facilitation skills to conduct group meetings and skills in recording meeting progress, attendance, participant measurements and problems or issues faced by participants.

The ASHAs residing in the villages randomised to the intervention, received training in 15 sessions over 5 days (Table 1). Training was provided by members of the research team. The instructional strategies used to deliver the training content included lectures, interactive and problem-based learning such as group discussions, role-play and case studies. Training was delivered using electronic slide presentations and flip charts. The flipcharts for participants are pictorially based, and there is one for each of the six meetings. ASHAs flipcharts have the same pictures as for participants, but every second sheet (on the back of the flipchart) includes text that the ASHA could use to educate participants (Figshare: https://figshare.com/s/7bbfcc22e0c9c91a5ca0). Video feedback was also used to demonstrate correct, and incorrect, measurement techniques. The classroom based training sessions took place close to the community at each of the three sites. ASHAs were compensated for the time they spent during the training, including travel expenses.

**Table 1:** Training agenda for ASHAs for the Control of Hypertension in Rural India Feasibility Study

Session	Title	Topics discussed
Day 1	Introduction	Roles and responsibilities of ASHAAims of CHIRI projectExpectations of research
	Pre-training test	Assessment of knowledge and skills
	Working with research group / research project	Expectation of data collection Need for consistency and accuracy
	Measurement training	Anthropometric measurement
	Prevention and control of Non-Communicable diseases (NCDs)	Modified from Ministry of Health and Family Welfare NCD ASHA training module number eight for prevention of NCDs [23]
Day 2	Measurement training	Measuring blood pressure and weight
	Goal setting	SMART Goals explanation and practice
	Hypertension knowledge	Community Meeting 1 Knowledge about high blood pressure, risk factors and complications
	Measurements	Measuring blood pressure and weight
Day 3	Review Goals & Problem-Solving	Review goals set previous day and problem solve if goals not achieved
	Self-care / management of hypertension	Community Meeting 2Recommendations for self-management, medication adherence, diet, physical activity, tobacco alcohol Referral to clinical care, monitoring etc.
	Physical Activity	Community Meeting 3Physical activity, recommended quantity, intensity and some practical and easy activities to practice
	Measurements	Measuring blood pressure and weight
	Goal Setting	Set another goal and complete action plan
Day 4	Review Goals & Problem Solving	Review goals set previous day and problem solve if goals not achieved
	Diet / Tobacco and Alcohol	Community Meeting 4Dietary approaches to prevent hypertension, tobacco control and alcohol cessation recommendations
	Practical self-management	Community Meeting 5Practical advice and support for self-management
	Measurements	Measuring blood pressure and weight
	Goal Setting	Goal setting and action plan
Day 5	Review Goals & Problem Solving	Review goals set previous day and problem solve if goals not achieved
	Putting it all together	Community Meeting 6Review key messages from the program
	Preparation	Preparing for monthly meetingsDealing with informational needsExpectation of the group
	Measurements	Measuring blood pressure and weight
	Review Goals & Problem Solving	Review goals set previous day and problem solve if goals not achieved
	Conclusion and final wrap up	Practicalities, (payment, who to contact, etc.), logistics
	Post-training test	Assessment of knowledge and skills

Following the training, the intervention (community-based education support groups) was implemented among community members who were identified as having hypertension during the baseline survey [19]. The main tasks for the ASHA in the intervention group was to encourage community members to attend, and lead the community meetings. For the research study, the project manager at each site supervised ASHAs and a research fellow observed meetings. Community meetings were held every 2 weeks over a three- month period by ASHAs who were paid an incentive to facilitate the meetings, on par with the Government of India standards. ASHAs measured blood pressure and weight of the attendees at the start of each session; led group-based learning about hypertension and its management; and assisted in promoting adoption of healthier lifestyles and setting SMART goals. All the ASHAs invited to participate in the study agreed and gave written informed consent in the local language; there was no penalty for refusal to participate.

Approval for the overall study was obtained from The Centre for Chronic Disease Control, India; Christian Medical College, Vellore, India; Sree Chitra Tirunal Institute for Medical Sciences and Technology, India; Health Ministry Screening Council, Ministry of Health and Family Welfare, India; Rishi Valley Ethics Committee, Rishi Valley School, Madanapalle, India; Monash University, Australia and Indian Council of Medical Research, Delhi, India.

### Evaluation

There were four components to the evaluation of the training program (please see Additional file 1: Table S2),

1. A knowledge and skills test was developed to assess the ASHAs level of knowledge and skills at baseline and after training (Figshare: https://figshare.com/s/b94c7af22ae220540c45). The test consisted of multiple choice and true/false type questions about hypertension, its risk factors, complications and healthy lifestyle and were based on previous research conducted by the team and existing literature [24, 25]. ASHAs at Rishi Valley and West Godavari had the same pre- and post-training test, while ASHAs at the Trivandrum site used a modified version of the test (Figshare: https://figshare.com/s/b94c7af22ae220540c45). The modification was required, as ASHAs from Trivandrum had received initial training for NCDs from the public health system.
2. A research officer from the study, who participated in the baseline survey, attended the meetings and assessed the performance of ASHAs using a monitoring and evaluation reporting sheets. The monitoring sheet comprised of a checklist of the tasks delivered, use of resources, time management and problem solving skills that ASHAs were trained for earlier. The evaluation forms are available online (Figshare: https://figshare.com/s/b94c7af22ae220540c45).
3. In addition to the written assessment, ASHAs were interviewed for 40–45 min in the local language to understand the major enablers and barriers in the implementation of the intervention. An external consultant, not involved in the training or the intervention implementation in all the sites, interviewed ASHAs. The interviewers used an interview guide to explore the perspectives of ASHAs about the training received. All the interviews were recorded, translated and transcribed into English. The interview guide is available online (Figshare: https://figshare.com/s/b94c7af22ae220540c45).
4. ASHAs were re-assessed for their knowledge and skills post-intervention using the same questionnaire at all three sites. (Figshare: https://figshare.com/s/b94c7af22ae220540c45).

**Fig. 1:**
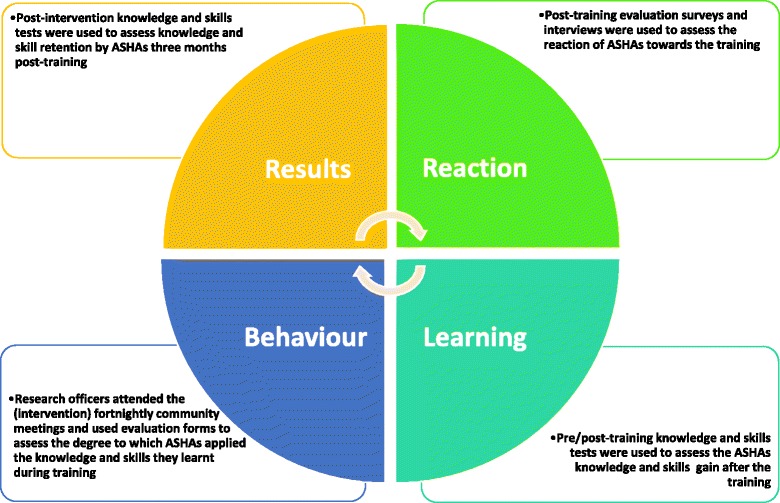
The Kirkpatrick’s evaluation of the ASHAs training

The effectiveness of training was based on Kirkpatrick’s four level evaluation model (Fig. 1) [18]:

- Learning: knowledge and skills achieved by trainees during training was assessed using the pre- (day 1) and post-training (day 5) tests;
- Result: the long-term outcomes of the training, i.e. the degree to which ASHAs could retain the knowledge and skills gained during training and apply it to the community, was evaluated using the post-intervention tests;
- Behaviour: the degree to which trainees applied the gained knowledge and skills during the intervention was assessed using forms, completed by research officers during the community education meetings, designed to monitor and evaluate the competence and performance of ASHAs; and
- Reaction: trainee’s perception and reactions toward the training was assessed in two ways. First, satisfaction of ASHAs with the training program was assessed at the end of training using evaluation forms that comprised both Likert-scale questions and open-ended questions. Secondly, at the end of the implementation period ASHAs were interviewed by external consultants about their experience of the training program and the implementation period. Training evaluation forms and interview guides are available online (Figshare: https://figshare.com/s/b94c7af22ae220540c45)

### Data analysis

Demographic characteristics of ASHAs were summarised using medians and ranges. Quantitative data on knowledge and skills was assessed using two-tailed paired t-tests for comparing scores within groups (SPSS version 23 for Windows). Qualitative data were obtained from training evaluation forms and research officers’ monitoring sheets. The forms comprised of structured and open-ended questions MA and RJ reviewed the evaluation forms and interview transcripts. The transcripts were manually coded and thematic analysis was conducted to determine ASHAs’ perceptions of the training, their community experience, challenges faced during the intervention and recommendations for the future. MA and RJ met to note any discrepancies and agree on the final data coding and emerging themes.

## Results

### ASHA participation

Eleven of the 15 ASHAs from the CHIRI intervention sites were appointed by the panchayat (local government), while four were recruited for the purposes of the study, due to vacant positions in the villages. Seven of these ASHAs, were recruited from West Godavari, and four each in Rishi Valley and Trivandrum. All the ASHAs attended all the training sessions and participated in the intervention.

The majority of the ASHAs were married (87%) and had completed at least 10 years of education (67%; Table 2). Their ages ranged from 19 to 50 years. The overall median age was 36 years but differed between sites, being least in Rishi Valley (29 years) and oldest in Trivandrum (40 years). Two thirds (60%) of the ASHAs had more than 6 years’ experience working in their communities, and worked between 2 and 6 h per day. In West Godavari, the mean work experience was 12 years, while that of the two other sites was 7 years. Furthermore, 73% had no other paid job other than their role as an ASHA. Eighty percent of the ASHAs had previously attended at least one training session provided by the NHRM, the public health training that was provided by the government, while almost half had attended all seven of these government training modules [23] . Fifty three percent of the ASHAs could communicate well in English. While all of them used cell phones, 47% of them shared the phone with their families (Table 2). Previous training received and other demographic variables did not seem to have an impact on the baseline knowledge of ASHAs.

**Table 2:** Demographics of ASHAs participating in the training program

	Rishi Valley	West Godavari	Trivandrum	Combined
Age (years)				
< 20	–	1 (13.0%)	–	1 (7.0%)
20–30	2 (50.0%)	2 (29.0%)	–	4 (27.0%)
30–40	2 (50.0%)	2 (29.0%)	2 (50.0%)	6 (40.0%)
> 40–50	–	2 (29.0%)	2 (50.0%)	4 (27.0%)
Median age	29	38	40	36
Education				
Class 10 and 11	4 (100%)	5 (71.0%)	1 (25%)	10(67.0%)
Class 12	–	2 (29.0%)	3 (75%)	5 (33.0%)
Knowledge of English				
Communicate well by reading and writing	1 (25.0%)	4 (56.5%)	3 (75.0%)	8 (53.0%)
Only read	2 (50.0%)	2 (29.0%)	–	4 (27.0%)
Do not communicate in English	1 (25.0%)	1 (14.5%)	1 (25.0%)	3 (20.0%)
Median age starting work as an ASHA (years)	21.5	21	31.5	23
Proportion of ASHAs having other paid duties*	1 (25.0%)	3 (43.0%)	–	4 (27.0%)
ASHA work experience (years)				
≤ 2	2 (50.0%)	2 (29.0%)	–	4 (27.0%)
3–6	1 (25.0%)	1 (14.5%)	–	2 (13.0%)
> 6	1 (25.0%)	4 (56.5%)	4 (100%)	9 (60.0%)
Mean work experience	7	12	7	9.5
ASHA usual working hours*				
< 2	2 (50.0%)	2 (29.0%)	–	4 (27.0%)
2–4	1 (25.0%)	1 (14.5%)	–	2 (13.0%)
4–6	–	3 (42.0%)	4 (100%)	7 (47.0%)
> 6	1 (25.0%)	1 (14.5%)	–	2 (13.0%)
Regular use of mobile phones	4 (100%)	7 (100%)	4 (100%)	15 (100%)
Proportion who share a phone with family members	2 (50.0%)	5 (71.0%)	–	7 (47.0%)
Ability to operate a smart phone	1 (25.0%)	1 (14.5%)	3 (75.0%)	5 (33.0%)

*Excludes unpaid household duties

### Learning outcomes

**Fig. 2:**
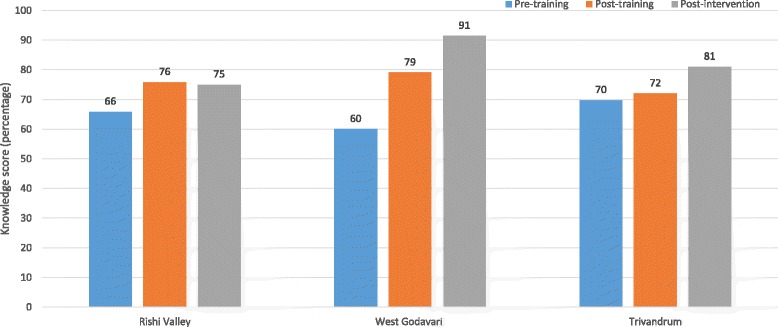
Pre-, Post-training and Post-intervention knowledge level change for ASHAs as part of the feasibility study of the CHIRI project

The mean knowledge score of all ASHAs at baseline was 64%, ranging from 60% in West Godavari to 70% in Trivandrum (Fig. 2). The mean overall knowledge score increased to 76% (t_14_ = 4.04, *p* ≤ 0.001) at the post-training assessment. Test scores and mean increase in knowledge score varied between the three sites. Only West Godavari showed a statistically significant increase of 19% (*p* ≤ 0.001) in the post-training test compared to the pre-training test.

### Changes in knowledge post-intervention

The post-intervention mean knowledge scores ranged from 75% in Rishi Valley to 91% in West Godavari (Fig. 2). The mean overall knowledge score was 84% (t_14_ = 4.65, *p* ≤ 0.001), demonstrating an increase of 20% from baseline and an 8% increase from post-training knowledge scores. Only West Godavari showed a statistically significant post-intervention increase of 12% (*p* = 0.02) compared to the post-training test scores.

### Behaviour

Using records documented by Research Officers during the community meetings we found that ASHAs led and facilitated the community meetings; they measured blood pressure and weighed the participants, and demonstrated and explained handouts and (Figshare: https://figshare.com/s/b94c7af22ae220540c45) as trained. They also helped the participants to set SMART goals and reviewed their action plan.

### Reaction

Interviews with ASHAs indicated that they had developed a better understanding about hypertension and had improved their skills in clinical anthropometric measurement (Table 3). They further conveyed that the training content was easy to understand and captured a wide range of relevant topics important for their job, including developing confidence in discussing hypertension within the community. The ASHAs appreciated the interactive training activities, which they reported had equipped them to understand real life scenarios and enabled them to overcome some of the challenges related to their new roles. For several ASHAs, the CHIRI training was the first time they were trained in measurement of blood pressure and weight, and they appreciated the opportunity to learn new skills. The ASHAs stated that the trainers were supportive and helped them understand their role as “Group supporters”. They reported that the flip charts and the pictures were useful in educating community members about risk factors for cardiovascular disease and adopting a healthy lifestyle. Most of the ASHAs used the knowledge gained to educate their families, neighbours and friends.

**Table 3:** Views and recommendations of ASHAs about the training

Training outcomes	
“Before I felt shy to talk, but now I’m more confident …” “It brought more interest to work” “Now it’s easy to motivate people” “I didn’t know much before training, after training I learnt lot of things … now I can give advice and help others” “It helped a lot; we learnt something new about health …. I learnt about blood pressure and how to control it” “The community members respect us because we are taking care of their health” “Our trainers taught us how to explain to people in understandable way and how to take decisions”	
Training material	
“Even though we explain things to them, it’s hard to get them to understand …. by seeing flip charts and images they can understand easily” “I went home and taught my family members” “It helped a lot, now I can check all the blood pressure of my family members, I’m more experienced and advise them to eat good food and exercise daily”	
Community experience and recommendations	
“People smile when they see us and keep telling us that they learnt a lot of things from us and they are taking good care of themselves now and doing more exercise. When we hear these words, we know they are interested to listen to us…. we get satisfied” “We should conduct meetings for young people so that they learn to take care of their heath” “They usually follow our advice, but soon they will forget. It’s better if we can set up scheduled visits to keep reminding them” “It will be good if you increase training period and should do more training on other health issues” “It is a very good program; it would be better to continue for 3 months we can learn more things”	
Challenges and difficulties	
“I have been working as an ASHA for 10 years, but my family is not happy with my job due to insufficient pay, they want me to quit” “Sometimes it takes us up to 5 months to receive our pay” “Some ASHAs went on strike but were told by our supervisors, that you will never get a fixed salary only incentives” “Housewives and farmers are busy, they don’t have time to come to the meetings”	

### Challenges and recommendations

Some of the challenges that were mentioned by the ASHAs in the interviews included a low response from community members, especially housewives and farmers who they perceived did not have sufficient time to attend group meetings. There was also concern about a lack of facilities for transportation to the meeting venue and failure to supply medicines during the community meetings. The topic of insufficient remuneration was raised by several ASHAs who reported that the incentives provided by the National Rural Health Mission were usually delayed. They reported a preference to be part of the health system and receive a regular salary. Some ASHAs also complained about the lack of supportive supervision and assistance from their line managers within the public health system.

ASHAs suggested the need for regular refresher training sessions. They also wanted to be trained on other chronic conditions such as diabetes mellitus. Some ASHAs suggested inviting young adults to their meetings so that they might be encouraged to adopt a healthy life style to prevent chronic diseases. Others suggested making monthly home visits for patients, to monitor hypertension and provide support in adhering to medications.

## Discussion

We have demonstrated that ASHAs can be trained to lead community-based group educational discussions, and support community members in the management of high blood pressure. We also showed that the methods and materials were amenable to each site, resulting in similar increases in knowledge of hypertension from baseline. The training program and training materials were appropriate for the different sites and no tailoring, except for the pre-and post-test questionnaire, was needed. The ASHAs were motivated and wanted to enhance their knowledge on other chronic diseases. The interviews suggested that the incentivisation (within government recommended salary structures) along with the aspiration to learn new skills and gain knowledge helped improve motivation levels, and practicing the skills each fortnight helped with the retention of knowledge over time.

Previous studies on the effectiveness of training CHWs for prevention and management of CVD and its risk factors, in low and middle income countries (LMICs), demonstrate immediate improvement post-training in knowledge among CHWs as well as practical skills [26–30]. Baseline scores improved between 3% and 40% after training and up to 35% after delivering the intervention. In two of these studies [28, 29] knowledge was retained for up to six [28] and 8 months [29]. Evaluation of the effectiveness of training was mostly done using pre-post training scores but two of the studies [28, 29] incorporated interviews and focus group discussions to capture the CHWs’ experiences of the training program. These are useful additions to the evaluation of the effectiveness of training using pre- and post-training tests as they enable assessment of both the motivation of trainees and their ability to implement the knowledge and skills gained in the work environment. Recent studies have provided evidence that behaviour change is necessary in implementation of evidence-based practice [31–33] which is usually the main aim of such training programs.

ASHAs were first engaged by the NRHM to be the primary interface between the community and public health system [15]. Their main role was to assist the Auxiliary nurse midwife (ANM) in providing maternal and child health related activities such as antenatal care and immunization. In some regions where the prevalence of NCDs is high, such as in the state of Kerala, ASHAs have been trained in prevention of NCDs and to provide support to the ANM in community-based preventive activities [34]. One of the challenges faced by ASHAs is that they are not employees of the health system and receive insufficient and sometimes delayed incentives for their work, which often translates, to poor motivation levels. ASHAs also reported feeling disadvantaged due to the lack of opportunities for advancement of their career. Similar findings were reported in another study where the lack of incentive and inappropriate career growth opportunities led to high turn-over rates and poor performance of CHWs [35].

In recent years, there have been several studies that have involved the training of CHWs to prevent and manage CVDs and risk factors in LMICs, while the authors are aware that a number of others are in progress [36, 37]. None of these studies has provided details about the effectiveness of the training or has published their training materials. With the expanding role of CHWs, which now also includes the prevention and management of NCDs, there is a need for effective training materials and methods to train them appropriately. At a health system level, it is important that CHWs have a defined job description, good supervision and appropriate remuneration. They should also have regular training and retraining which is evaluated and supported by appropriate supervision.

### Strengths and limitations

The primary strength of this study is the use of the Kirkpatrick’s four level evaluation model [18] to assess not only the effectiveness of the training program in changing knowledge scores, but also in the assessment of behaviour change, motivation and reaction to the training. Like most feasibility studies, our main limitation is the relatively small number of ASHAs included and the relatively short duration of the intervention. A statistically significant improvement was detected in West Godavari (seven ASHAs) only. There were trends for improvement in the other two sites, so given the small sample sizes; the absence of a detectable effect could reflect type II error. Moreover, the relatively poor baseline levels of knowledge in West Godavari may have resulted in a larger effect size. We did not assess the knowledge level of ASHAs in the control group, and so are unable to exclude other factors that may have influenced knowledge. Nevertheless, we developed a culturally adapted CVD training program for ASHAs, and have made these materials publicly available for others to use (at Figshare). Most LMICs have a health workforce similar to ASHAs, such as Barangay health workers in Philippines, Lady health workers in Pakistan, and Shastho Shebikas in Bangladesh. These countries face similar challenges in terms of disease burden and access to healthcare [38] and hence, could potentially use culturally adapted training strategies and the available workforce to increase health-care access for managing hypertension.

## Conclusions

In summary, we demonstrate that training ASHAs for management of hypertension is feasible and leads to change in knowledge, skills and motivation. Our findings emphasise the need for culturally appropriate training materials for NCDs and their risk factors, which can be delivered using interactive and innovative methods. To get a better picture of the effectiveness of training, using frameworks such as the Kirkpatrick model [18] is important; since these models do not focus on a single outcome measure and emphasise the importance of using multiple measures for the evaluation of training, thereby allowing more comprehensive comparison and interpretation of training outcomes. Use of these models also draws attention to the learning transfer process and the behavioural change of the trainee to achieve the desired outcome of the training [39]. Future studies might be best focussed on assessment of the appropriate time and methods for re-training. In the era of technology, where most of the ASHAs had access to mobile phones, technology could also potentially be used for re-training or sending reminders. Use of these devices could also reduce the cost and time of training [40] and improve the overall performance and quality of care provided [41]. Changes in the health system, such as career opportunities for ASHAs, performance based incentives delivered on time, and innovative training techniques would help improve the morale of this rural community based health workforce.

## Additional file

Additional file 1: **Table S1.** Description of Training Materials and Sessions. All downloadable from https://figshare.com/s/7bbfcc22e0c9c91a5ca03 DOI: https://doi.org/10.4225/03/5967f9a94970d. ASHA Training Manual. **Table S2.** Evaluation Materials and ASHA Resources. All downloadable from https://figshare.com/s/b94c7af22ae220540c45 DOI: https://doi.org/10.4225/03/5975a0f9da160. (DOC 78 kb)

## Abbreviations

ANM: Auxiliary Nurse Midwife
ASHA: Accredited Social Health Activists
CHIRI: Control of Hypertension In Rural India
CHW: Community Health Worker
CVD: Cardiovascular disease
LMIC: Low and Middle Income Country
NCDs: Non-Communicable Diseases
NPCPCS: National Program for the Prevention and Control of Cardiovascular Diseases, Diabetes, Cancer and Stroke
NRHM: National Rural Health Mission
