# Supplementary material for: Evaluation of a training program of hypertension for accredited social health activists (ASHA) in rural India
Source: BMC Health Serv Res. 2018 May 2;18:320. doi: 10.1186/s12913-018-3140-8 (PMC5932780; doi:10.1186/s12913-018-3140-8)
Supplement: Supplementary file 1 — Table S1. Description of Training Materials and Sessions. All downloadable from https://figshare.com/s/7bbfcc22e0c9c91a5ca03 DOI: https://doi.org/10.4225/03/5967f9a94970d. ASHA Training Manual. Table S2. Evaluation Materials and ASHA Resources. All downloadable from https://figshare.com/s/b94c7af22ae220540c45 DOI: https://doi.org/10.4225/03/5975a0f9da160. (DOC 78 kb) [file 12913_2018_3140_MOESM1_ESM.doc]

**Table S1. Description of Training Materials and Sessions. All downloadable from** [**https://figshare.com/s/7bbfcc22e0c9c91a5ca0**](https://figshare.com/s/7bbfcc22e0c9c91a5ca0)

**DOI: 10.4225/03/5967f9a94970d**

| **Document Title** | **Training & Meeting** | **Description** |
| --- | --- | --- |
| ASHA_Manual_final_14_July_2017_with_acknowledgements.pdf | Training | Training manual for health workers |
| Meeting_1_ASHA_flip_chart_July_2017.pdf | 1 | Flipchart for ASHAs to use at Meeting 1 |
| Meeting_1_Goal_setting_flip_chart_July_2017.pdf | Training & Meeting 1 | Flipchart to use to teach ASHAs about Goal Setting. This is also used by ASHAs in Meeting 1 to teach individuals how to set their goals |
| Meeting_1_Particpant_flip_chart_July_2017.pdf | 1 | Flipchart for participants to use at Meeting 1 |
| Meeting_2_ASHA_flip_chart_July_2017.pdf | 2 | Flipchart for ASHAs to use at Meeting 2 |
| Meeting_2_Participant_flip_chart_July_2017.pdf | 2 | Flipchart for participants to use at Meeting 2 |
| Meeting_2_Participant_handouts_2.1_2.2_combined.pdf | 2 | Handouts to be provided to participants at the end of Meeting 2 |
| Meeting_3_ASHA_flip_chart_July_2017.pdf | 3 | Flipchart for ASHAs to use at Meeting 3 |
| Meeting_3_Participant_flip_chart_July_2017.pdf | 3 | Flipchart for participants to use at Meeting 3 |
| Meeting_3_participant_handout_3.6_goal_setting_sheets_July_2017.pdf | 3 | Handout to be provided to participants at the end of Meeting 3 |
| Meeting_4_ASHA_flip_chart_July_2017.pdf | 4 | Flipchart for ASHAs to use at Meeting 4 |
| Meeting_4_Participant_flip_chart_July_2017.pdf | 4 | Flipchart for participants to use at Meeting 4 |
| Meeting_4_Participant_handout_1_2_3_4_combined.pdf | 4 | Handouts to be provided to participants at the end of Meeting 4 |
| Meeting_5_ASHA_flip_cart_July_2017.pdf | 5 | Flipchart for ASHAs to use at Meeting 5 |
| Meeting_5_Participant_flip_cart_July_2017.pdf | 5 | Flipchart for participants to use at Meeting 5 |
| Meeting_5_Participant_Handout_5.1_Action_Plan.pdf | 5 | Handout to be provided to participants at the end of Meeting 5 |
| Meeting_5_Participant_handout_5.2_Weekly_self_management_diary.pdf | 5 | Weekly Self-Management Diary to be provided to participants at the end of Meeting 5 |
| Meeting_6_ASHA_flip_chart_July_2017.pdf | 6 | Flipchart for ASHAs to use at Meeting 6 |
| Meeting_6_Participant_flip_chart_July_2017.pdf | 6 | Flipchart for participants to use at Meeting 6 |
| Meeting_6_Participant_handout_6.1_to_6.8 | 6 | Handouts to be provided to participants at the end of Meeting 6 |
| ASHA_Meeting_resources_final_14_07_2017_with_acknowledgements.pdf | All | Resource manual used during training and each meeting with patients. |

**Table S2. Evaluation Materials and ASHA Resources. All downloadable from** [**https://figshare.com/s/b94c7af22ae220540c45**](https://figshare.com/s/b94c7af22ae220540c45)

**DOI: 10.4225/03/5975a0f9da160**

| **Document Title** | **Description** |
| --- | --- |
| ASHA_Meeting_resources_final_14_07_2017_with_acknowledgements.pdf | Resource manual used during training and each meeting with patients. It includes all questionnaires to be used for evaluation (except for the others listed here) |
| CHIRI_Pre_training_Knowledge_evaluation_ASHA_HCP.pdf | Questionnaire administered to ASHAs prior to training (Godavari and Rishi Valley) |
| Kerala_ASHA_Pre_training_test and Post_training_test.pdf | Questionnaire administered to ASHAs before and after training at the Kerala site |
| CHIRI_Post_training_knowledge_test_and_evaluation_ASHA.pdf | Questionnaire administered to ASHAs after training (Godavari and Rishi Valley) |
| Research Officer Monitoring and Evaluation Sheet | Form used by Research Officers to evaluate the ASHAs’ performance during the intervention |
| CHIRI_Post_intervention_knowledge_test_and_evaluation_ASHA.pdf | Questionnaire administered to ASHAs after completion of the intervention (all sites) |
| CHIRI_Post_Intervention_Focus Group Discussion_Interview_Guide_ASHA.pdf | Focus Group Discussion questions administered to ASHAs after completion of the intervention (all sites) |
